# Mutual fitness benefits arise during coevolution in a nematode‐defensive microbe model

**DOI:** 10.1002/evl3.58

**Published:** 2018-05-28

**Authors:** Charlotte Rafaluk‐Mohr, Ben Ashby, Dylan A. Dahan, Kayla C. King

**Affiliations:** ^1^ Department of Zoology University of Oxford Oxford OX1 3PS United Kingdom; ^2^ Department of Mathematical Sciences University of Bath Bath BA2 7AY United Kingdom; ^3^ Integrative Biology University of California Berkeley Berkeley California 94720; ^4^ Current Address: Department of Microbiology and Immunology Stanford University School of Medicine Stanford California 94305

**Keywords:** *C. elegans*, coevolution, defensive mutualism, *E. faecalis*, experimental evolution, host–parasite interaction, microbiota, *S. aureus*

## Abstract

Species interactions can shift along the parasitism‐mutualism continuum. However, the consequences of these transitions for coevolutionary interactions remain unclear. We experimentally coevolved a novel species interaction between *Caenorhabditis elegans* hosts and a mildly parasitic bacterium, *Enterococcus faecalis*, with host‐protective properties against virulent *Staphylococcus aureus*. Coinfections drove the evolutionary transition of the *C. elegans–E. faecalis* relationship toward a reciprocally beneficial interaction. As *E. faecalis* evolved to protect nematodes against *S. aureus* infection, hosts adapted by accommodating greater numbers of protective bacteria. The mutualism was strongest in pairings of contemporary coevolved populations. To generally assess the conditions under which these defensive mutualisms can arise and coevolve, we analyzed a model that showed that they are favored when mild parasites confer an intermediate level of protection. Our results reveal that coevolution can shape the transition of animal‐parasite interactions toward defensive symbioses in response to coinfections.

**Impact Summary**

Many animal and plant species harbor beneficial microbes that protect them from parasite attack. Despite the widespread nature of these defensive mutualisms, little is known about how these interactions arise and whether they are coadapted and shaped by reciprocal selection between host and microbe populations. Knowledge of such processes is important for determining how defensive mutualists might drive changes in parasite infection outcomes in nature. There are also potential applications for this information if host and microbe genetic background could impact the effectiveness of microbe‐conferred protection, particularly during probiotic use or bacteriotherapy (i.e., faecal microbiome transplants).

In this study, we artificially coevolved a nematode host and bacteria species in the lab to address these ideas. We find that although first interactions between host and resident bacterium are mildly parasitic, the resident evolved over time to become a highly beneficial protector when other, more virulent parasites attacked its host. The host reciprocally evolved to accommodate more colonization by defensive bacteria, despite the costs involved in the partnership. We also show that these mutualisms can be coadapted as the benefits to both species (protection for host, within‐host colonization for bacterium) was greatest in combinations of coevolved host‐defensive bacterium populations at the same point in evolutionary time. Accompanying mathematical theory further expands on the conditions under which defensive mutualisms can arise and coevolve, from initially parasitic relationships. Here, we highlight the importance of both host and microbe evolution in the formation of defensive mutualisms.

Microbes are not simply passengers or parasites, but can confer beneficial traits to their hosts with important consequences for community interactions. In particular, microbial symbionts can determine host susceptibility to parasites in the environment through defensive mutualism. Recognized for over a century (Belt 2002), defensive mutualism has been observed across plant (Mendes et al. 2011; May and Nelson 2014) and animal species (Dillon et al. 2005; Dong et al. 2009; Jaenike et al. 2010; Koch and Schmid‐Hempel 2011), including humans (Kamada et al. 2013). Given the ubiquity and negative fitness consequences of parasites (Pedersen and Fenton 2007), here referring to organisms gaining fitness benefits from host exploitation, microbial symbionts that confer protection greatly benefit hosts as an additional line of defense.

Many mutualistic host‐microbe relationships are hypothesized to have been formed through long‐term coevolutionary interactions (Shoemaker et al. 2002; Ochman et al. 2010; Sanders et al. 2014). Microbial symbiont phylogenies often strongly correlate with those of their hosts (Shoemaker et al. 2002; Quek et al. 2004; Ley et al. 2008; Ochman et al. 2010; Kwong et al. 2014; Sanders et al. 2014), and symbionts typically perform suboptimally in novel host species (e.g. McGraw et al. 2002). To date, most studies of host‐microbe mutualisms are focused on long‐established interactions (Douglas 1998; Nyholm and McFall‐Ngai 2004). While coevolution has the potential to reinforce strength in mutualisms (Thrall et al. 2007), it is unclear how the evolution of mutualism is initiated.

For coevolving defensive mutualisms, in which host and microbial symbiont receive mutual benefits, the answer to their adaptive origin and coadaptation may lie in the wider community context. Host and defensive symbiont interactions can straddle the parasitism‐mutualism continuum (Betts et al. 2016), being costly for their hosts to carry, but providing a net benefit upon parasite attack (Hughes et al. 2011; Vorburger and Gouskov 2011; Ford and King 2016). In nature, parasite species are rarely found infecting hosts in isolation, but are often in coinfections with other parasite species (Telfer et al. 2010) and in coinfection, can provide inadvertent benefits to their hosts, for example, by modulating the virulence of competing parasite species (Gardner et al. 2004; Selva et al. 2009). These context‐dependent relationships are evolvable (King et al. 2016; Ashby and King 2017). King et al. (2016) found that mildly virulent parasites can evolve rapidly within nematode hosts to protect against more virulent parasites, though still remain costly when virulent competitors are absent. However, the consequences of this evolutionary transition on reciprocal host evolution are unknown. Hosts may be less likely to evolve resistance to parasites in the absence of a defensive symbiont. In *Drosophila* evolved in the presence of a parasite with and without a defensive symbiont, alleles protecting against infection were under weaker selection in those individuals with the defensive symbiont (Martinez et al. 2016). Alternatively, hosts may evolve to actively select for mutualists among their microbiota (McLoughlin et al. 2016).

Here, we investigated the adaptive emergence and coevolution of defensive mutualism using a novel tripartite system. We experimentally copassaged nematode hosts (*Caenorhabditis elegans*) and a costly bacterium (*Enterococcus faecalis*) for 14 host generations in communities varying in the presence of a more virulent parasite (*Staphylococcus aureus). E. faecalis* was previously shown to evolve rapidly to protect its nonevolving nematode host against virulent infection by *S. aureus* (King et al. 2016). Both treatments consisted of five replicate populations started from a single clone of *E. faecalis* and a genetically diverse nematode population. We found that *S. aureus* infection during coevolution drove enhanced microbe‐mediated protective effects for hosts, and reciprocally, higher within‐host colonization for *E. faecalis*. These outcomes, which simultaneously benefit both host and defensive microbe, were strongest in pairings of contemporary time‐points, indicating the mutualism became increasingly coadapted over evolutionary time. Using a general mathematical model, we asked under what conditions defensive mutualisms can arise, and specifically when would hosts evolve reduced resistance to the costly symbiont. We simulated the conditions under which two parasites infect a host, one of which conveys protection to the other. Our analysis revealed that defensive mutualisms readily coevolve when the strength of protection is intermediate given the context‐dependent nature of the relationship. Together, our experimental and theoretical results indicate that host‐parasite coevolutionary interactions can readily transition into an interaction that is mutually beneficial in communities with a shared enemy.

## Methods

### SYSTEM

We use the *C. elegans*–*E. faecalis*–*S. aureus* laboratory‐based experimental system. *E. faecalis* and *S. aureus* can both act as parasites to *C. elegans*. Under our experimental conditions *E. faecalis* is only mildly pathogenic toward *C. elegans*, but *S. aureus* induces higher levels of host mortality (Ford et al. 2016; King et al. 2016). *E. faecalis* colonizes *C. elegans* guts (King et al. 2016; Ford et al. 2017) and provides protection to *C. elegans* hosts against *S. aureus* via the production of superoxides that directly attack *S. aureus* cells within the host (King et al. 2016).

### COEVOLUTION EXPERIMENT

The *C. elegans* line EEVD00, generated by the lab of Henrique Teotonio (UEN, Paris, France), was used to start the coevolution experiment. Aliquots of the ancestral population were frozen at the start of the experiment to allow them to be revived when needed for phenotypic assays. This line is a genetically diverse, obligately outcrossing, dioecious population, encompassing the genetic diversity of 16 geographically diverse natural nematode isolates (Theologidis et al. 2014). Ancestral *C. elegans* were initially grown up on nematode growth medium (NGM) on 9 cm petri plates seeded with *E. coli* OP50 food bacteria. A portion of these worms were immediately frozen in buffer (20% DMSO) at –80°C to create a static frozen stock of ancestral worms.

**Figure 1:**
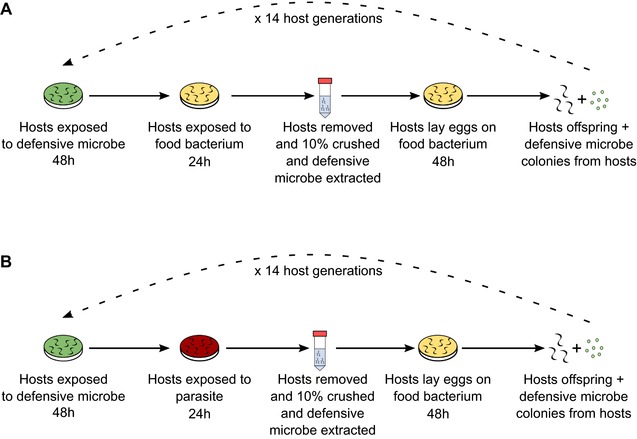
Schematic of the evolution experiment. Figure 1A represents the COEV‐P treatment and 1B the COEV+P treatment. Both nematodes and defensive microbes were passaged to the next generation in both treatments. There were five replicate populations of each treatment and evolution was carried out for 14 host generations.

**Figure 2:**
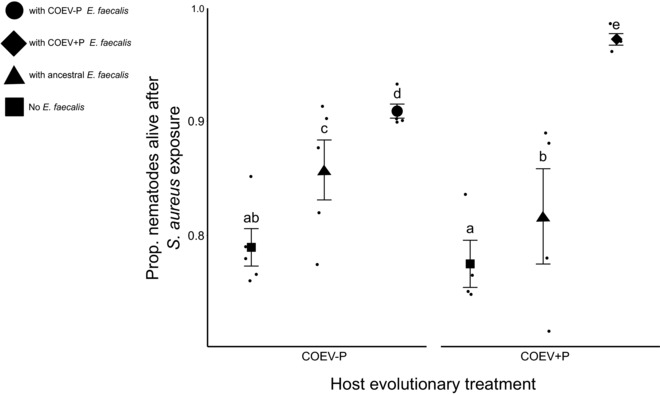
Survival of *C. elegans* from each coevolution treatment after *S. aureus* parasite exposure, following precolonization by sympatric, coevolved *E. faecalis*, ancestral *E. faecalis* or *E. coli* OP50 food. Means with the same letter do not differ significantly from one another (Tukey multiple comparisons). Squares represent treatment means. Dots represent means for each replicate. Error bars, ±1 SEM.

The evolution experiment consisted of two treatments (1) *C. elegans* was copassaged with *E. faecalis* alone (COEV‐P treatment) (Fig. 1A), and (2) *C. elegans* was copassaged with *E. faecalis* in the presence of genetically fixed *S. aureus* parasite (MSSA 476), under which conditions *E. faecalis* protects its host (King et al. 2016) (COEV+P treatment) (Fig. 1B). Both treatments followed the same basic protocol (Fig. 1). Initially, plates of ancestral *C. elegans* containing gravid females were “bleached” using a sodium hypochlorite solution to surface sterilize eggs and synchronize the population (Stiernagle 2006). As an additional synchronization step, sterilized eggs were suspended in 7 mL of M9 buffer in 15 mL centrifuge tubes and incubated overnight at 20°C, shaking continuously on an orbital shaker at 88 rpm. Under these conditions, eggs hatch but arrest at the L1 larval stage (Stiernagle 2006). Simultaneously, the *E. faecalis* strain OG1RF (Garsin et al. 2001) was cultured overnight at 30°C from a single colony in Todd Hewitt Broth (THB) and OP50 cultured overnight at 30°C in Luria‐Bertani broth (LB). A portion of the *E. faecalis* overnight culture was frozen in 25% Glycerol at –80°C to maintain a static ancestral stock. For each treatment, five 9 cm NGM plates were inoculated, each with 300 μL OP50 and an equal volume of *E. faecalis* culture, and dried at room temperature for 30–60 min., to represent five replicate populations for each treatment. Approximately 2000 L1 worms were added to each plate, which were then dried for a further 60 min at room temperature before being incubated at 20°C for 48 h. This mimics a horizontally transmitted, bacterial symbiont becoming resident in the host during early development. Meanwhile the *S. aureus* strain MSSA476 (Holden et al. 2004) was grown up from a frozen stock overnight at 30°C in THB and OP50 cultured overnight at 30°C in LB. The following day, ten Tryptic Soy Broth (TSB) Agar plates were prepared, five plates were inoculated with 100 μL of *S. aureus* overnight culture and the remaining plates inoculated with 100 μL of OP50 overnight culture. The plates were incubated overnight at 30°C. After 48 h exposed to *E. faecalis* plates, nematodes in the COEV‐P treatment were transferred to OP50, while worms in the COEV+P treatment were transferred to *S. aureus* exposure plates. Worms were washed four times (Jansen et al. 2015) to remove *E. faecalis* from the nematode cuticle. After transfer, worms were left exposed to the *S. aureus* parasite or OP50 food for 24 h at 25°C.

*Enterococcus faecalis* and worms were then copassaged. To passage *E. faecalis* after parasite or OP50 exposure, worms were washed again (Jansen et al. 2015), but resuspended in 250μl M9 buffer, and 10% of each suspension of a population was manually crushed with a plastic pestle. Crushed worm suspension was spread on selective media (TSB agar with 100 μg/mL rifampicin) to isolate *E. faecalis* strain OG1RF. An *E. faecalis* overnight culture was grown for each population by picking 100 colonies from the streaked out rifampicin TSB plate and then grown in THB at 30°C overnight and an *E. coli* OP50 grown up at 30°C in parallel. The remainder of the washed, alive worms from each population was transferred to an OP50‐seeded NGM plate for 48 h to lay eggs. Subsequently, nematode eggs were again bleached and synchronized overnight in M9 buffer. The cycle was then completed as described above and repeated for a total of 14 host generations of coevolution (Fig. 1).

### *E. faecalis* PROTECTIVE ABILITY TOWARD *C. elegans*

Protective ability of *E. faecalis* was measured as host survival following *E. faecalis* preexposure and subsequent *S. aureus* parasite exposure. All survival assays were carried out over the time frame of one generation of the evolution experiment and with approximately 200 worms for each replicate population. Archived replicate populations were thawed onto OP50 food‐seeded NGM plates five days prior to the assay, to revive L1 stage worms (Stiernagle 2006) and allow them to reach adulthood and lay eggs. Assay plates were prepared as described above, but on 6 cm petri dishes with the following proportions of bacteria: 200 μL OP50/200 μL *E. faecalis* population, 400 μL OP50 culture (for OP50‐only controls), and 60 μL *S. aureus* culture. Exposures were either with sympatric, end‐point coevolved worms and *E. faecalis* or ancestral, archived stock of one player, with evolved end‐point strains of the other. Worms were exposed to *E. faecalis* for 48 h followed by *S. aureus* for 24 h. Following final exposure, numbers of alive and dead worms were counted on the *S. aureus* plates.

### QUANTIFYING *E. faecalis* ACCUMULATION IN NEMATODE GUT

Replicate populations of ancestral and coevolved nematodes and *E. faecalis* were prepared as described above. Bacteria were allowed to accumulate in nematodes by exposing generation 14 worms to their coevolved *E. faecalis* replicate population (sympatric combination) and ancestral *C. elegans* with generation 14 *E. faecalis* populations (allopatric combinations), from the COEV‐P and COEV+P treatments. Approximately, 200 L1 nematodes were added to each plate and left for 48 h at 20**°**C. Five female *C. elegans* per replicate were picked and their cuticle rinsed in M9 buffer, following which, nematodes were manually crushed with pestles to release their gut bacteria. Gut contents were plated on selective media (TSB with 100 μg/mL rifampicin) and incubated at 30°C overnight. *E. faecalis* colony‐forming units (CFUs) were counted.

### STATISTICAL ANALYSIS OF EXPERIMENTAL DATA

Survival data were analyzed with nested binomial mixed effect models (GLMMs), followed by Tukey multiple‐comparison tests (R package multcomp) to determine pairwise differences. CFU data were log‐transformed and analyzed using a nested linear model‐mixed effects model followed by pairwise *t*‐tests. The false discovery rate (FDR) correction was used to correct for multiple testing where appropriate. All statistical analyses were carried out in R version 3.2.3.

### MATHEMATICAL MODEL

To assess the conditions under which defensive mutualism can evolve, we analyze the eco‐evolutionary dynamics of a general model of host protection (Ashby and King 2017), extended to allow coevolution (see Table 1 for full list of parameters). The model consists of a host and two infectious species, one of which may convey protection against the other. The defensive mutualist (akin to *E. faecalis*) is mildly virulent and can evolve to protect its host by reducing susceptibility to a more virulent parasite (akin to *S. aureus*). The theoretical model is not intended to mimic the experiments, but rather is a broadly applicable model of mutualism coevolution in a generic system.

**Table 1:** Parameters for the theoretical model

Parameter/variable	Description
***S***, ***I***_***j***_, ***I***_***MP***_, *** N***	Number of hosts that are: susceptible, infected by species *j*, infected by both species, alive (total)
***a***	Maximum per‐capita host birth rate
$\tilde{b},b\left(x\right)$	Host natural mortality rate: baseline, trade‐off for hosts with strategy *x*
$c_{j}^{1}$	Strength of trade‐off for species *j*
$c_{j}^{2}$	Shape of trade‐off for species *j*
***f***_***j***_, ***f***_***MP***_	Relative fecundity for hosts infected by: species *j*, both species
***q***	Density‐dependence coefficient
$\alpha_{j},\;\alpha_{M\;P}$	Additional mortality rate for hosts infected by: species *j*, both species
${\tilde{\beta }}_{j}$	Baseline transmission rate for species *j*
***β***_***M***_(***x***, ***y***)	Transmission rate for defensive mutualists with strategy *y* when hosts have strategy *x*
***β***_***P***_(***y***)	Transmission rate for parasites when hosts are infected by defensive mutualists with strategy *y*
***γ***_***j***_	Recovery rate for hosts infected by species *j*
***λ***_***M***_(***x***, ***y***)	Force of infection for the defensive mutualist when hosts and defensive mutualists have strategies *x* and *y*, respectively
$\lambda_{P,S},\lambda_{P}\left(y\right)$	Force of infection for the parasite when hosts are: susceptible, already infected by defensive mutualists with strategy *y*
***ν***	Host birth rate: $\nu =\left(a-qN\right)\left(S+f_{M}I_{M}+f_{P}I_{P}+f_{\mathrm{MP}}I_{\mathrm{MP}}\right)$
***x***	Host susceptibility strategy to protective parasite/defensive mutualist
***y***	Strength of protection conferred to the host

For simplicity, we assume that co‐infections only occur between parasites of different species (previous work has shown that the evolution of host protection is broadly similar if this assumption is relaxed; Ashby and King 2017). The host population is therefore divided into four classes according to its infection status: susceptible to both species (*S*); infected by the defensive mutualist but still susceptible to the parasite $\left(I_{M}\right)$; infected by the parasite but still susceptible to the defensive mutualist $\left(I_{P}\right)$; and infected by both species $\left(I_{\mathrm{MP}}\right)$. Hosts have a base natural mortality rate of $\tilde{b}$ and reproduce at a maximum per‐capita rate of *a* subject to density‐dependent competition (defined by qN with $N=S+I_{M}+I_{P}+I_{\mathrm{MP}}$) and reduced fecundity (*f*) when infected $\left(0\leq f_{M},f_{P},f_{\mathrm{MP}}\leq 1\right)$, giving a birth rate of $\nu =\left(a-qN\right)\left(S+f_{M}I_{M}+f_{P}I_{P}+f_{\mathrm{MP}}I_{\mathrm{MP}}\right)$. The maximum pairwise transmission rate for species *j* is ${\tilde{\beta }}_{j}$ and recovery occurs at rate $\gamma_{j}$. Hosts experiencing a single infection with species *j* suffer an additional mortality rate (virulence) of $\alpha_{j}$, while mixed infections lead to an additional mortality rate of $\alpha_{\mathrm{MP}}=\alpha_{M}+\alpha_{P}$. The virulence of the parasite is assumed to be higher than the additional mortality caused by the defensive mutualist $\left(\alpha_{P}>\alpha_{M}\right)$.

We investigate the evolution of two traits: (1) host susceptibility to the defensive mutualist, denoted by strategy x≥0, and (2) resistance conferred to the host by the defensive mutualist, denoted by strategy *y* (0≤y≤1). In the absence of the parasite, infection by the defensive mutualist leads to increased mortality (i.e., the defensive mutualist is a mildly virulent parasite). However, if the more virulent parasite is present in the population, then infection by the defensive mutualist may reduce the risk of subsequent infection. Specifically, $\beta_{P}\left(y\right)={\tilde{\beta }}_{P}\left(1-y\right)$, which means that host susceptibility decreases with y>0.

Conveying protection to the host is likely to be costly for the defensive mutualist, as it must divert resources from growth or reproduction to bolster host defenses. We therefore set$\;\beta_{M}\left(x,y\right)={\tilde{\beta }}_{M}\left(1-c_{M}\left(y\right)\right)\left(x+1\right)$, where $c_{M}\left(y\right)=c_{M}^{1}\left(1-e^{c_{M}^{2}y}\right)/\left(1-e^{c_{M}^{2}}\right)$ controls the trade‐off (i.e., the reduction in onward transmission of the defensive mutualist due to conveying host protection). The parameter $c_{M}^{1}$ ($0\leq c_{M}^{1}\leq 1)$ determines the maximum strength of the cost (reduction in transmission), and $c_{M}^{2}\neq 0$ determines the shape of the trade‐off, with $c_{M}^{2}>0$ implying that costs of conveying host protection accelerate, and $c_{M}^{2}<0$ implying that the associated costs decelerate with greater host protection. Positive values of *x* indicate that the host is actively helping the defensive mutualist by increasing its susceptibility. Similarly, hosts may pay a cost of increased susceptibility in the form of a higher natural mortality rate, $b\left(x\right)=\tilde{b}\left(1+c_{H}\left(x\right)\right)$, where $\tilde{b}$ is the base natural mortality rate and $c_{H}\left(x\right)=c_{H}^{1}x^{c_{H}^{2}}$ is the host trade‐off. Again, the parameter $c_{H}^{1}\geq 0$ controls the overall strength of the trade‐off (i.e., the proportional increase in the mortality rate) and $c_{H}^{2}>0$ modifies that shape of the trade‐off such that costs accelerate when $c_{H}^{2}>1$ and decelerate when $0<c_{H}^{2}<1$. We include the potential for an explicit cost to hosts associated with increasing susceptibility to the defensive mutualist (i.e., when x>0). This is because the host may also inadvertently increase its susceptibility to other infections not captured by the model. When $c_{H}^{1}=0$ there is no explicit cost to the host associated with increased susceptibility to the defensive mutualist.

Assuming monomorphic, well‐mixed populations, the epidemiological dynamics are fully described by the following set of differential equations:

(1a) $$\frac{\mathrm{dS}}{\mathrm{dt}}=\nu -\left[b\left(x\right)+\lambda_{M}\left(x,y\right)+\lambda_{P,S}\right]S+\gamma_{M}I_{M}+\gamma_{P}I_{P}$$

(1b) dIMdt=λMx,yS−bx+αM+γM+λPyIM+γPI MP

(1c) dIPdt=λP,SS−bx+αP+γP+λMx,yIP+γMI MP

(1d) dI MP dt=λMx,yIP+λPyIM−bx+αMP+γM+γPI MP

where $\lambda_{M}\left(x,y\right)=\beta_{M}\left(x,y\right)\left(I_{M}+I_{\mathrm{MP}}\right)$, $\lambda_{P}\left(y\right)=\beta_{P}\left(y\right)\left(I_{P}+I_{\mathrm{MP}}\right)$ and $\lambda_{P,S}={\tilde{\beta }}_{P}\left(I_{P}+I_{\mathrm{MP}}\right)$ are the forces of infection for the defensive mutualist, the parasite on hosts infected with the mutualist, and the parasite on uninfected hosts, respectively.

We explore the coevolutionary dynamics of this system using evolutionary invasion analysis, which assumes traits are continuous, selection is weak, and there is a separation of ecological and evolutionary timescales (Geritz et al. 1998). This means that traits are governed by many loci with small additive effects, mutations are rare, and mutants are phenotypically similar to the resident population. We analyze the model numerically because there is no analytic expression for the epidemiological equilibrium of the system. We relax the assumptions of rare mutations and weak selection in our simulations by coarsely discretizing the interval for the strategies (larger mutations, stronger selection) and by introducing mutants before the system reaches equilibrium (no separation of timescales). Starting with a single resident trait in each population, $\left(x_{r},y_{r}\right)$, we solve the ODE system for a given time period [0, *T*] (T=100), then randomly introduce a mutant at low frequency in one population (xm=xr±εH or ym=yr±εM)or (mutation sizes fixed at $\varepsilon_{H}=\varepsilon_{M}=0.02$), We then rerun the ODE solver over the period [T,2T] and remove any strains that have fallen below a frequency of $\varepsilon_{\mathrm{EXT}}=10^{-3}$. If more than one trait is still present in the population, then the next mutant is chosen based on a weighted probability of the trait frequencies. The process is repeated for n=2000 iterations. The source code for the simulations is available in the online supplementary material.

## Results

### EXPERIMENTAL RESULTS

Experimental evolution strongly impacted microbe‐mediated host protection. We found a significant interaction of nematode host and *E. faecalis* evolutionary background (COEV+P vs COEV‐P treatment) on microbe‐ mediated host survival (Nested binomial GLMM: χ^2^ = 109.8, d.f. = 2, *P* = <0.001, Fig. 2). The interaction between sympatric host‐defensive mutualist pairings resulted in the highest level of host survival after *S. aureus* infection in the COEV+P treatment (Fig. 2), with hosts surviving 5% better in this treatment than in COEV‐P sympatric pairings, which resulted in the next highest level of host survival (Fig. 2). The effect of preexposure alone (Defensive mutualist evolutionary background) was significant (Nested binomial GLMM: χ^2^ = 384.7, d.f. = 2, *P* = <0.001) but host evolutionary background alone was not (Nested binomial GLMM: χ^2^ = 0.7, d.f. = 1, *P* = 0.422).

**Figure 3:**
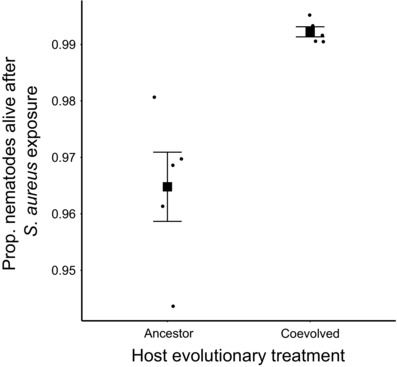
Survival of ancestral and coevolved *C. elegans* after *S. aureus* parasite exposure, following precolonization by coevolved *E. faecalis*. Squares represent treatment means. Dots represent means for each replicate. Error bars, ±1 SEM.

Examining how hosts evolved, coevolution of *E. faecalis* with nematodes under *S. aureus* attack resulted in significantly enhanced protection toward coevolved, sympatric hosts in the COEV+P treatment in comparison to COEV+P bacteria paired with ancestral hosts (Nested binomial GLMM: χ^2^ = 27.4, d.f. = 1, *P* = <0.001, Fig. 3), with COEV+P defensive mutualists increasing the percentages of hosts surviving parasite attack from just over 96% to near 100% (Fig. 3). Coevolution did not result in a statistically significant increase in microbe‐mediated host survival for ancestral *C. elegans*, although there was a trend in the direction of COEV+P bacteria protecting better than COEV‐P or ancestral bacteria (Nested binomial GLMM: χ^2^ = 3.6, d.f. = 2, *P* = 0.163, Fig. S1).

**Figure 4:**
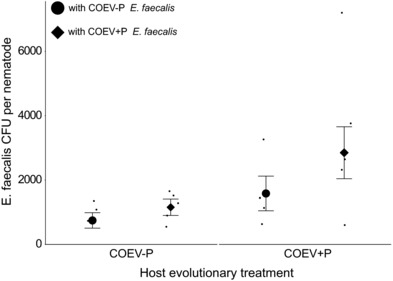
Gut colonization by coevolved *E. faeaclis* of ancestral and sympatric, coevolved *C. elegans* hosts. Error bars, ±1 SEM.

There was a significant effect of both *E. faecalis* (Nested linear‐mixed effects model: χ^2^ = 3.8, d.f. = 1, *P* = 0.049) and *C. elegans* (Nested linear‐mixed effects model: χ^2^ = 3.8, d.f. = 1, *P* = 0.016) background on *E. faecalis* accumulation/nematode (Fig. 4), with COEV+P *E. faecalis* colonizing its sympatric, coevolved worms significantly better than any other defensive mutualist‐host combination (Fig. 4). Colonization in the coevolved sympatric treatment combination was 1.5X greater than in COEV+P host with COEV‐P parasite pairings, which showed the next highest colonization levels (Fig. 4). There was no significant interaction between *E. faecalis* and *C. elegans* effects (Nested linear‐mixed effects model: χ^2^ = 0.004, d.f. = 1, *P* = 0.947). Pairwise *t*‐tests revealed, however, that the significant host and defensive mutualist effects were caused entirely by heightened colonization in the sympatric COEV+P host‐defensive mutualist combination in comparison to COEV‐P worms exposed to COEV‐P defensive mutualists (*P* = 0.017). The enhancement of the COEV+P host‐defensive mutualist combination in both colonization (Fig. 4) and protection (Fig. 2) suggests these traits are linked. There was no significant difference in *S. aureus* accumulation in the gut among nematode strains (Nested linear‐mixed effects model: χ^2^ = 1.2, d.f. = 2, *P* = 0.544, Fig. S2).

### THEORETICAL RESULTS

We first consider host evolution with a static defensive mutualist. In the supplementary material, we derive an expression for host fitness in the special case when there is no recovery $\left(\gamma_{M}=\gamma_{P}=0\right)$ and hosts infected by the parasite do not reproduce $\left(f_{P}=f_{\mathrm{MP}}=0\right)$, as these assumptions greatly simplify the expression for host fitness (eq. S2).

**Figure 5:**
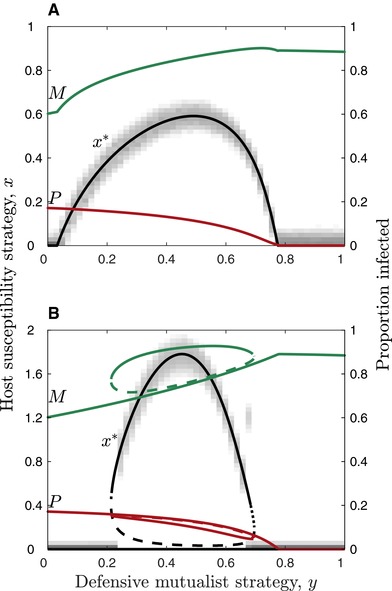
Evolution of host susceptibility to the defensive mutualist (black) for fixed levels of conferred protection. Host susceptibility to the defensive mutualist and the level of conferred protection increase with x and y, respectively. (A) Accelerating host trade‐off $\left(c_{H}^{2}=2\right)$; (B) decelerating host trade‐off $\left(c_{H}^{2}=0.5\right)$. Green and red curves show the equilibrium proportion of hosts infected with the defensive mutualist (M) and the parasite (P), respectively. Black curves show the singular strategy $\left(x^{*}\right)$ for the host: solid curves correspond to continuously stable strategies, dashed curves to evolutionary repellers and dotted curves to evolutionary branching points where two host types may evolve and coexist from an initially monomorphic population. Shading corresponds to simulation outputs, where the adaptive dynamics assumptions of weak selection and rare mutations are relaxed. Hosts infected by the parasite cannot recover or reproduce (see Fig. S3 for the converse). Fixed parameters: a=1, b=0.5, $c_{H}^{1}=0.02$, $c_{M}^{1}=0.1$, $f_{M}=1,$ $f_{P}=0,$ $f_{\mathrm{MP}}=0$, q=0.5, $\alpha_{M}=0.01$, $\alpha_{P}=1$, ${\tilde{\beta }}_{M}=5$, ${\tilde{\beta }}_{P}=5$, $\gamma_{M}=0$, $\gamma_{P}=0$.

Our analysis reveals that the host maximizes susceptibility to the defensive mutualist for intermediate levels of host protection (*y*) (Fig. 5). When *y* is small, the defensive mutualist only confers weak protection against the virulent parasite, which is insufficient to offset the associated costs of harboring the defensive mutualist. When *y* is large, the defensive mutualist confers strong protection that reduces the prevalence of the parasite in the population and hence the risk of infection. It is therefore only for intermediate values of *y* that the host evolves increased susceptibility to the defensive mutualist. These results are consistent when the host does not experience a trade‐off ($c_{H}^{1}=0)$, hosts infected by the virulent parasite can recover or reproduce (Fig. S3), and as the shape of the host trade‐off is varied from accelerating (Fig. 5A, S3A) to decelerating (Fig. 5B, S3B). When the trade‐off decelerates, intermediate levels of protection usually lead to hosts evolving either high or low susceptibility depending on the initial conditions and mutation size (i.e., the singular strategy is a repeller), but for a narrow range of parameters an initially monomorphic host population may diversify into two coexisting strategies through disruptive selection (i.e., the singular strategy is a branching point). The simulations, which relax the adaptive dynamics assumptions of weak selection and rare mutants, closely match our numerical predictions (Fig. 5, S3).

**Figure 6:**
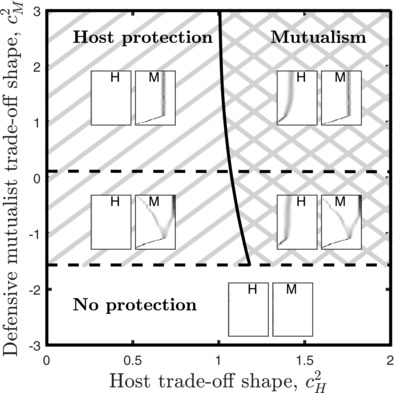
Qualitative coevolutionary outcomes as the shape of the host and defensive mutualist trade‐offs are varied. When $c_{H}^{2}>1$ the host trade‐off accelerates and when $c_{H}^{2}<1$ it decelerates. When $c_{M}^{2}>0$ the defensive mutualist trade‐off accelerates and when $c_{M}^{2}<0$ it decelerates. Host protection $\left(x^{*}=0,\;y^{\;*}>0\right)$ evolves in the single hatched region, and mutualism $\left(x^{*}>0,\;y^{\;*}>0\right)$ evolves in the crosshatched region. For trade‐offs between the two horizontal dashed lines, the defensive mutualist diversifies into two strains, one conferring high protection to the host and the other conferring no protection. The inset figures show simulations corresponding to the different regions, with the host (H) and defensive mutualist (M) traits (*x* and *y*) increasing from left to right in each plot, and time increasing from bottom to top. Mutations are small and initially x=0 and y=0 (the relationship starts off as being antagonistic). Hosts infected by the parasite cannot recover or reproduce (see Fig. S4 for the converse). Fixed parameters as in Figure 5, with $c_{H}^{1}=0.02$ and $c_{M}^{1}=0.1.$

We now consider coevolution between the host and the defensive mutualist (Fig. 6, S4–S5). This scenario is analogous to the experiments, where both the host (*C. elegans*) and a mildly virulent bacteria (*E. faecalis*) are coevolved in the presence of a more virulent parasite (*S. aureus*). We focus on the case where infected hosts do not recover or reproduce, and assume that mutations are small and that initially x=0 and y=0 (the relationship starts off as being antagonistic). Since the host only evolves increased susceptibility when the protective parasite/defensive mutualist confers intermediate protection against the virulent parasite, we know that $x^{*}>0$ implies $y^{*}>0$ (“mutualism”). This tends to occur when the trade‐off for the host is relatively weak (small $c_{H}^{1}$, Fig. S5) and accelerates ($c_{H}^{2}>1$, Fig. 6), and when the trade‐off for the defensive mutualist is of intermediate magnitude (moderate $c_{M}^{1}$, Fig. S5) and does not strongly decelerate (Fig. 6). Alternatively, the defensive mutualist can evolve to protect the host even when the host does not reciprocate by increasing its susceptibility to the defensive mutualist (“host protection,” $x^{*}=0$, $y^{*}>0$). This generally occurs when the trade‐off for the host is relatively costly (large $c_{H}^{1}$, Fig. S5) or decelerating ($c_{H}^{2}<1$, Fig. 6), and when the trade‐off for the defensive mutualist is not too high (low to moderate $c_{M}^{1}$, Fig. S5) and does not strongly decelerate (Fig. 6). In both the “mutualism” and “host protection” cases, weakly decelerating or accelerating trade‐off shapes for the defensive mutualist can lead to evolutionary branching and the coexistence of high and low protective strains ($c_{M}^{2}\approx 0$, Fig. 6). For strongly decelerating trade‐offs $\left(c_{H}^{2}\ll 1,c_{M}^{2}\ll 0\right)$ host protection does not evolve, and so the relationship remains antagonistic. The results are broadly similar when infected hosts are allowed to recover or reproduce, although evolutionary branching is slightly less likely (Fig. S4). In summary, the model predicts that mutualism can readily evolve provided host protection is intermediate and is most likely to occur when the host trade‐off accelerates and the defensive mutualist trade‐off is not strongly decelerating.

## Discussion

Many beneficial symbioses are thought to have been formed through longstanding coevolutionary associations (Sanders et al. 2014). However, it is unclear how mutualisms arise and are shaped by coevolution from initially novel and even parasitic interactions. By combining an experimental coevolution approach with a theoretical model, we examined the de novo formation of a reciprocal host‐microbe defensive mutualism. Consistent with previous findings (King et al. 2016), *E. faecalis* here evolves to cross the parasitism‐mutualism continuum, becoming a host‐protective mutualist during coinfection with *S. aureus*. Nevertheless, we further find that *E. faecalis* reciprocally benefits, with higher within‐host fitness, and is best at protecting its sympatric coevolved host populations. Increased protection may directly result from the higher within‐host fitness within sympatric species interactions. Protection toward sympatric, coevolved hosts is greater than the cumulative effects of general increased protection by defensive microbes toward ancestral hosts and general increased survival of hosts with ancestral defensive microbes. This result is consistent with hosts evolving higher susceptibility to their sympatric defensive microbes. Our mathematical model confirms that host adaptation to defensive microbes can involve lower levels of genetic‐based resistance to these mutualists, provided they are not too costly and they confer at least an intermediate level of protection. These are both assumptions that reflect the biology of our tripartite model system (King et al. 2016).

Although mutualism coevolution has been studied in natural systems (e.g., De Mazancourt et al. 2005; Thompson 2005, 2014), experimental demonstrations are rare and/or under one‐sided adaptation conditions (Bracewell and Six 2015; Jansen et al. 2015; King et al. 2016; Morran et al. 2016), whereby the microbe evolves with a static host population (Rafaluk et al. 2015). Furthermore, previous studies have generally focused on broad phenotypic outcomes (e.g., Jousselin et al. 2003; Machado et al. 2005), rather than specific host and microbe effects important for understanding the patterns and processes of coevolution. That hosts here evolved increased susceptibility to their sympatric *E. faecalis* over time is consistent with de novo host adaptation via symbiosis, whereby host colonization by a defensive symbiont is selected for as a defense against parasite attack (Jaenike et al. 2010). This result reflects some defensive symbioses found naturally (Schmid et al. 2012), for example in North American fruit flies where hosts harboring *Spiroplasma* bacteria receive protection from a sterilizing nematode parasite (Jaenike et al. 2010). Strong main effects of both host and defensive microbe on the accumulation of *E. faecalis* indicate that worm hosts evolved to allow for increased defensive microbe colonization throughout the process of coevolution, despite their costs.

The fitness benefits for both defensive microbe and hosts increased over evolutionary time. Moreover, sympatric, coevolved pairings show the highest host survival and defensive mutualist colonization levels, relative to those from mismatched pairings in time. These results indicate some degree of coadaptation between mutualists, a finding not always present in defensive mutualisms. For example, in an aphid‐symbiont system, coadaptation occurs in the interaction between defensive microbe and parasite (Rouchet and Vorburger 2012; Parker et al. 2017), but not with the host (Parker et al. 2017). In systems where strong host‐symbiont coadaptation exists, it is when there are phylogenetic concordance and/or the symbiont is inherited with millions of years of association (Shoemaker et al. 2002; Jousselin et al. 2003; Quek et al. 2004; Wade 2007). However, it is increasingly known that many inherited bacteria can also transmit horizontally across host lineages (Parratt et al. 2016), and mechanisms of coadaptation have been well‐characterized in horizontally transmitted microbe‐host mutualisms, such as between squid and *Vibrio fischeri* (McFall‐Ngai and Ruby 1991; Nyholm and McFall‐Ngai 2004; Nyholm and Nishiguchi 2008; Collins et al. 2012). Yet, the evolutionary processes driving coadaptation, whether it exists beyond the species‐level across populations, and the time periods under which it can arise all remain elusive. In the present study, it took 14 host generations at most for heightened protection to result from a novel host‐microbe interaction. This effect was consistent across all replicate populations. These data reveal the potential for a mutualistic interaction to arise rapidly, and in a parallel fashion, in coevolving mutualisms with horizontally transmitted bacteria. From an applied perspective, these findings might be encouraging for the rapid establishment and success of novel host‐defensive microbe associations being used to stop transmission of devastating human parasites, such as *Zika* (Aliota et al. 2016) and dengue virus from insect vectors (Bull and Turelli 2013).

Our theoretical model shows that such evolutionary outcomes can occur across a range of fitness trade‐offs, and thus may be common in nature. Specifically, where relatively mild parasites show intermediate levels of protection against more virulent competitors in coinfection, selection drives these parasites toward defensive mutualism. Consistent with this theoretical conclusion, the selective environment involving *S. aureus* parasites resulted in increases of *E. faecalis*‐mediated protection, and also higher within‐host microbe fitness. These mutual benefits were also the result of host—*E.faecalis* coadaptation, as benefits were strongest in contemporary, sympatric pairings. Previously, this system has been used to show that under one‐sided adaptation conditions (King et al. 2016), *E. faecalis* can evolve to increasingly benefit a single genotype of *C. elegans* hosts. Here, we go beyond this finding. We show that under coevolutionary conditions with a genetically diverse host population, enhanced protection evolves as a consequence of evolutionary change in both host and mutualist. Our results ultimately reveal that mutualistic host‐microbe relationships can arise quickly and stably coevolve, and exhibit some degree of coadaptation across populations over time.

## CONFLICT OF INTEREST

We have no conflicts of interest.

Associate Editor: A. Gardner

## Supporting information


**Figure S1**. Survival of ancestral *C. elegans* after *S. aureus* parasite exposure, following pre‐colonisation by ancestral or coevolved *E. faecalis*.
**Figure S2**. Gut colonisation by *S.aureus* in ancestral and coevolved nematode populations.
**Figure S3**. Evolution of host susceptibility to the defensive mutualist (black) for fixed levels of conferred protection.
**Figure S4**. Qualitative coevolutionary outcomes as the shape of the host and defensive mutualist trade‐offs are varied (hosts infected by the parasite can recover and reproduce).
**Figure S5**. Quantitative coevolutionary outcomes for (A, C) hosts and (B, D) defensive mutualists as the strength of the cost functions vary.
