# Supplementary material for: Mutual fitness benefits arise during coevolution in a nematode‐defensive microbe model
Source: Evol Lett. 2018 May 28;2(3):246–56. doi: 10.1002/evl3.58 (PMC6121859; doi:10.1002/evl3.58)
Supplement: Supplementary file 1 — Figure S1. Survival of ancestral C. elegans after S. aureus parasite exposure, following pre‐colonisation by ancestral or coevolved E. faecalis. Figure S2. Gut colonisation by S.aureus in ancestral and coevolved nematode populations. Figure S3. Evolution of host susceptibility to the defensive mutualist (black) for fixed levels of conferred protection. Figure S4. Qualitative coevolutionary outcomes as the shape of the host and defensive mutualist trade‐offs are varied (hosts infected by the parasite can recover and reproduce). Figure S5. Quantitative coevolutionary outcomes for (A, C) hosts and (B, D) defensive mutualists as the strength of the cost functions vary. [file EVL3-2-246-s001.zip › evl358-sup-0001-SuppMat.docx]

**Supplementary material**

**Theoretical model: host evolution**

We first derive an expression for host fitness in the special case when there is no recovery and hosts infected by the parasite do not reproduce , as these assumptions greatly simplify the expression for host fitness. The initial dynamics of a rare host mutant () in a monomorphic resident population at equilibrium (*) are then given by:

Using the next-generation method (Hurford *et al.* 2010), one can derive the following expression, which is sign equivalent to the fitness of the mutant:

where and . The selection gradient for the host is then given by :

where

The population will evolve in the direction of the selection gradient until an evolutionarily singular strategy, , is reached at . The singular strategy is *evolutionarily stable* (ES; i.e. a local fitness maximum) if and is *convergence stable* (CS; i.e. locally attracting) if for and for (Geritz *et al.* 1998). If satisfies both criteria then it is a *continuously stable strategy* (CSS). If is CS but not ES, then it is an evolutionary branching point, and if it is neither CS nor ES then it is a repeller.

**Theoretical model: defensive mutualist evolution**

Following (Ashby & King 2017), it can be shown that the invasion fitness of a rare defensive mutualist (with ) is sign equivalent to

where and . The selection gradient for the defensive mutualist, , is then given by:

Using equations S3 and S6, we can find the co-singular strategies of the system (i.e. when both equations are simultaneously equal to 0) and numerically determine evolutionary stability. We verify the quantitative and qualitative outcomes through simulations.

**Supplementary methods**

*Quantifying S. aureus accumulation in host gut*

Nematodes from the evolved and ancestral populations were bleached as described above and synchronised in M9 buffer overnight. L1 larvae were grown up on 9cm NGM plates seeded with OP50 food for 48 hours, after which they were transferred to 9 cm *S. aureus* TSB plates, prepared as described above. Worms were exposed to *S. aureus* for 24 hours, following which they were filter tip washed as described above, crushed and gut contents plated onto MSA plates to select for *S. aureus* colonies only and incubated overnight at 30oC. *S. aureus* CFUs were counted.

**References**

Ashby, B. & King, K.C. (2017). Friendly foes: The evolution of host protection by a parasite. *Evol. Lett.* 1:211–221.

Geritz, S.A.H., Kisdi, E., Meszena, G. & Metz, J.A.J. (1998). Evolutionarily singular strategies and the adaptive growth and branching of the evolutionary tree. *Evol. Ecol.* 12:35–37.

Hurford, A., Cownden, D. & Day, T. (2010). Next-generation tools for evolutionary invasion analyses. *J. R. Soc. Interface* 7:561–571.


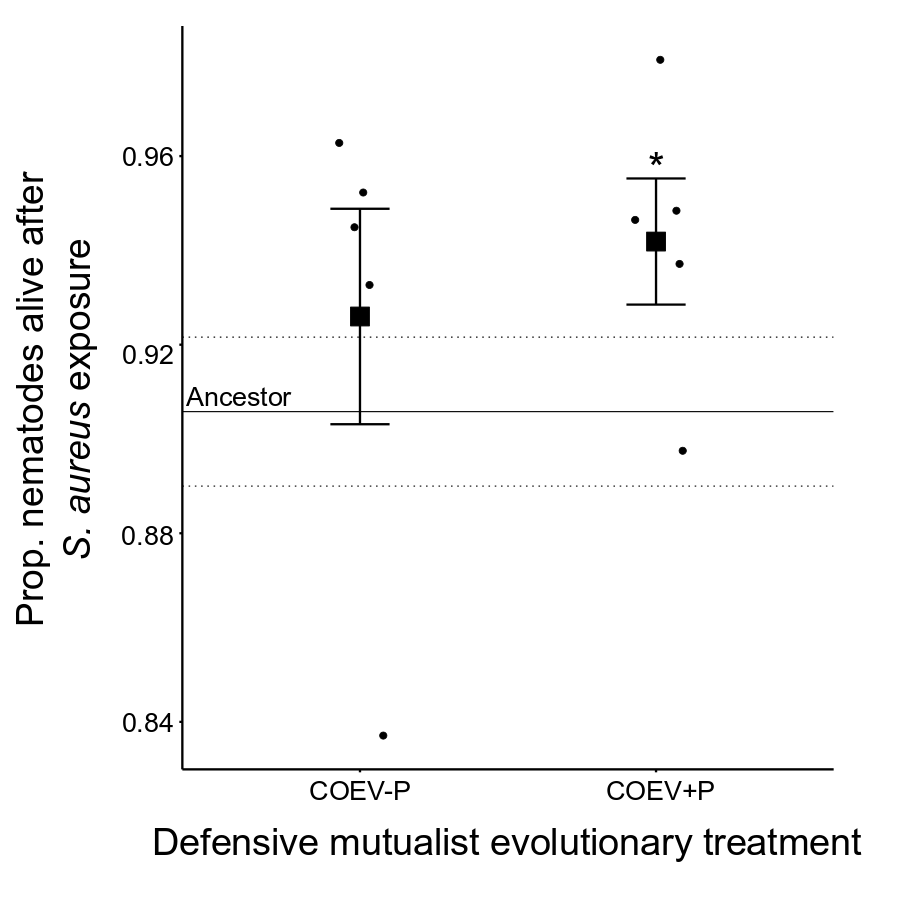


**Figure S1.** Survival of ancestral *C. elegans* after *S. aureus* parasite exposure, following pre-colonisation by ancestral or coevolved *E. faecalis*. Means marked with an asterisk differ significantly from the ancestor (Tukey contrasts). Error bars, ±1 S.E.M.


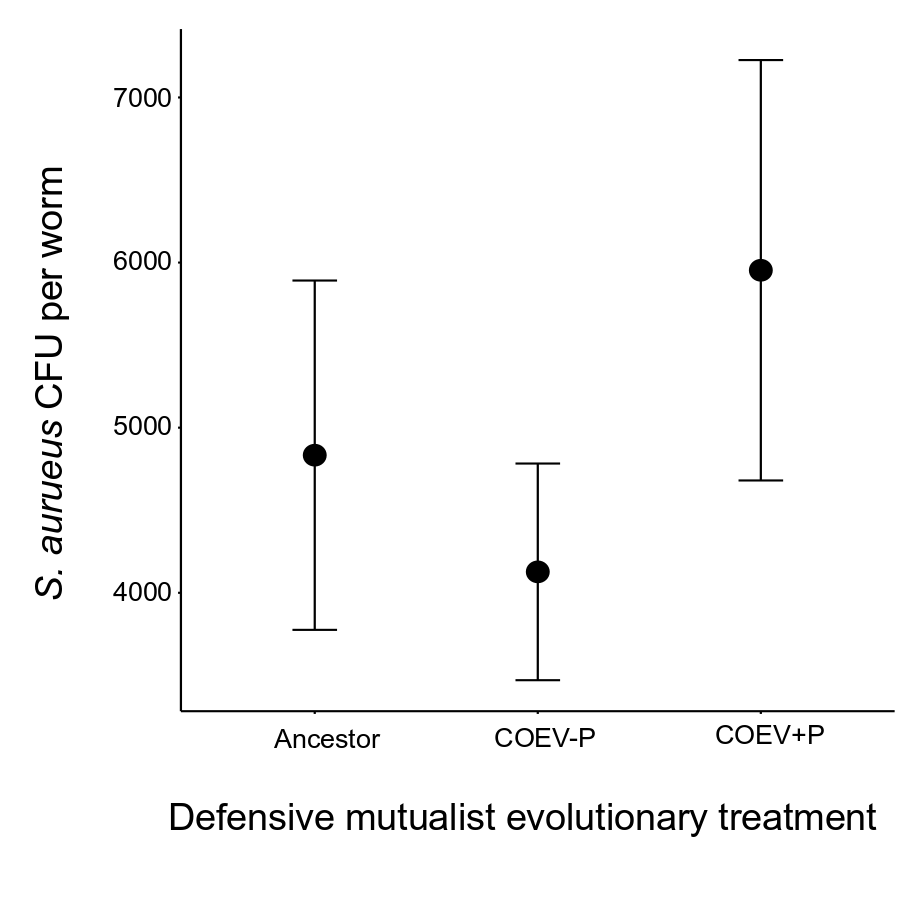


**Figure S2.** Gut colonisation by *S.aureus* in ancestral and coevolved nematode populations.

**Figure S3.** Evolution of host susceptibility to the defensive mutualist (black) for fixed levels of conferred protection. Host susceptibility to the defensive mutualist and the level of conferred protection increase with and , respectively. (A) accelerating host trade-off ; (B) decelerating host trade-off . Green and red curves show the equilibrium proportion of hosts infected with the defensive mutualist (M) and the parasite (P), respectively. Black curves show the singular strategy for the host: solid curves correspond to continuously stable strategies, dashed curves to evolutionary repellers and dotted curves to evolutionary branching points where two host types may evolve and coexist from an initially monomorphic population. Shading corresponds to simulation outputs, where the adaptive dynamics assumptions of weak selection and rare mutations are relaxed. Hosts infected by the parasite can recover and reproduce. Fixed parameters as in Fig. 5, except: , , .

**Figure S4.** Qualitative coevolutionary outcomes as the shape of the host and defensive mutualist trade-offs are varied (hosts infected by the parasite can recover and reproduce). When the host trade-off accelerates and when it decelerates. When the defensive mutualist trade-off accelerates and when it decelerates. Host protection evolves in the single hatched region, and mutualism evolves in the crosshatched region. For trade-offs between the two horizontal dashed lines, the defensive mutualist diversifies into two strains, one conferring high protection to the host and the other conferring no protection. The inset figures show simulations corresponding to the different regions, with the host (H) and defensive mutualist (M) traits ( and ) increasing from left to right in each plot, and time increasing from bottom to top. Mutations are small and initially and (the relationship starts off as being antagonistic). Fixed parameters as in Figure S3, with and

**Figure S5.** Quantitative coevolutionary outcomes for (A, C) hosts and (B, D) defensive mutualists as the strength of the cost functions vary. The host trade-off accelerates in the top row and decelerates in the bottom row . In cases where an evolutionary repeller exists (for decelerating host trade-offs), we show the non-zero value of the trait that evolves. Fixed parameters as in Figure 5, with .
